# Therapeutic effects of Iranian herbal extracts against *Trichomonas vaginalis*

**DOI:** 10.18869/acadpub.ibj.21.5.285

**Published:** 2017-09

**Authors:** Zohreh Fakhrieh Kashan, Mahdi Delavari, Mohsen Arbabi, Hossein Hooshyar

**Affiliations:** 1 Department of Medical Parasitology, School of Public Health, Tehran University of Medical Sciences, Tehran, Iran; 2 Department of Medical Parasitology, School of Medicine, Kashan University of Medical Sciences, Kashan, Iran

**Keywords:** *Trichomonas vaginalis*, Medicinal plants, Iran, Herbal, Extract

## Abstract

*Trichomonas vaginalis* is a flagellated parasite affecting about 276 million people annually worldwide. Tricomoniasis is associated with different complications in pregnant women and infants. 5’-nitroimidazole derivatives (metronidazole, ornidazole, and tinidazole) are FDA approved drugs recommended for trichomoniasis treatment. Treatment with metronidazole 5’-nitroimidazole derivatives is associated with many side effects, and drug resistance to metronidazole has been reported in some cases. Recently, many attempts have been made to evaluate the effects of plants on causative agents of vaginal infections. In our research, the national and international databases were searched and the effects of various herbal extracts on *T. vaginalis* in Iran were reviewed from 2006 to 2016. In articles investigated, some plants had favorable antitrichomonal effects and needed to be further investigated. All the plant extracts have only been evaluated *in vitro*. Surveys of different articles in this review show that the active ingredients present in different parts of plants, including aerial parts, leaves, flowers, stems, and root can be suitable sources for introducing and developing new antitrichomonal compounds

## INTRODUCTION

*Trichomonas vaginalis* is a protozoan parasite and the causative agent of infection in human vagina, prostate gland, and urethra[1-3]. *T. vaginalis* is a parasite of the Trichomonadidae family and genus of Trichomonas. Morphologically, this parasite is pyriform and occasionally amoeboid form[3-6]. Prevalence of trichomoniasis is estimated about 276 million new cases annually worldwide and associated with different complications such as preterm birth, high mortality in infant, and low birth weight[7-9]. About 85% of infected women and 77% of infected men with this parasite are asymptomatic. In men, trichomoniasis can be associated with urethral discharge and dysuria. In general, among women, the common sites of infection are vagina, urethra, and endocervix, and clinical features include vaginal discharge (which is often diffuse, malodorous, and yellow-green), dysuria, itching, pruritis, vulvar irritation, and abdominal pain. Other complications could be infection of the adnexal, endometrium, and Skene’s and Bartholin’s glands. Trichomoniasis also may cause the increased risks of other sexually transmitted diseases; infection is associated with 1.5–2.7 times greater risks of HIV acquisition and transmission[10-13]. Trichomoniasis in women is more common at the age of 16-53. Infection is usually transmitted by sexual contact and restricted to the genital tract. Wet mount examination is the most common method of diagnosis, but *T. vaginalis* infection can also be diagnosed by clinical signs, cultures (TYM, TYI-S-33, dorset, and horse serum), and molecular approaches such as polymerase chain reaction, which is the most sensitive and specific method for the detection of *T. vaginalis*[14-16]. The appropriate treatment for trichomoniasis includes the 5’-nitroimidazole derivatives of which metronidazole and tinidazole are the only efficient FDA approved drugs. Recently, some other drugs have been introduced for the treatment of this disease (Table 1)[17-29]. Besides, simultaneous treatment of both sexual partners (female and male) is recommended[17-20]. Treatment efficiency depends on the influence of host factors. Nitroimidazole may also be required for the effective treatment of HIV-infected women who are infected with *T. vaginalis*. Drug resistance to metronidazole has been reported in some cases[30,31]. In recent years, many attempts have been made for the evaluation of the effects of plants on different microorganisms that cause vaginal infection on *T. vaginalis* in vaginal infection. Nowadays, many studies have been performed in replacing medicinal plants with synthetic drugs[32-36].

**Table 1:** Chemotherapeutic agents against trichomoniasis

Drug name	Medial use	Ref.
Metronidazole	Anti-protozoan (giardiasis, trichomoniasis, and amebiasis)	[17-20]
Tinidazole	Anti-protozoan (amebiasis, giardiasis, and trichomonasis)	[21,22]
Secnidazole	Anti-protozoan (trichomonasis and dientamoebiasis)	[21,29]
Ornidazole	Anti-protozoan (trichomoniasis)	[23]
Nimorazole	Anti-protozoan (trichomoniasis and head and neck cancer)	[24]
Nitrothiazole	Anti-protozoan (giardiasis, trichomoniasis, and amebiasis)	[25]
Nitazoxanide	Anti-protozoan (cryptosporidiosis, giardiasis, hymenolepiasis, ascariasis, and resistant Trichomoniasis)	[26,25]
Hamycin	Anti-protozoan (trichomoniasis)	[27]
Paramomycin	Cutaneous leishmaniasis, visceral leishmaniasis, and anti-recurrent *T. vaginalis*	[28]
Sodium nitrite	Anti-microbial and anti-*T. vaginalis* resistance to metronidazole (used in traditional medicine)	[29]
Nitrofuran	Antimicrobials and anti-*T. vaginalis*	[29]
Furazolidone	Antibacterial and anti-*T. vaginalis*	[29]

Ref., references

Search of electronic databases and journals was performed to find the related data reporting the *in vitro* effects of various plant extracts on *T. vaginalis* in Iran. The international databases, including PubMed, Scirus, ISI Web of Science, Scopus, EMBASE, Science Direct, and Google Scholar, as well as national databases covering Iran Medex, IranDoc, Magiran, and Scientific Information Database (ISC) were searched. These searches limited to articles published between the years 2006 and 2016. The keywords used for the literature search for this review were “*Trichomonas vaginalis*”, “medicinal plants”, “herbal drug”, “plant extract”, “anti-trichomonas activity”, “*in vitro*”, and “Iran”.

### Chemical drugs for treatment of trichomoniasis

#### Metronidazole

Metronidazole is a member of the nitroimidazole family and prevents genetic material synthesis by disrupting the DNA of microrganism cells. Metronidazole is highly active against anaerobic bacteria and anaerobic parasites including *T. vaginalis*[30,37]. The pharmacological properties of this drug are suitable, and it is accessible as oral, intravenous, vaginal, and topical forms. After oral administration, it is well absorbed and reached the highest level within 1 to 2 hours after ingestion[30,37,38]. This drug is effective against many anaerobic bacteria and parasites. Urinary tract is the prominent way of metronidazole excretion. Anaerobic infections, such as abdominal infections, genital tract infections, septicemia, endocardial infection, bone infections, brain and spinal cord infections, pulmonary infections, cutaneous infections. Before some surgeries, metronidazole is used to prevent secondary infections[30,37,38].

#### Side effects and the problem of metronidazole resistance

Side effects in systemic metronidazole therapy (in less than 1% of patients) include vomiting, diarrhea, abdominal cramps, headache, drowsiness and fever. Intravenous administration is typically associated with thrombophlebitis. Infrequent side effects consists mouth ulcers, headache, glossitis, dizziness, inflammation of the mouth and lips and dark-colored urine[17-29,39]. Irregular and long-term use of the metronidazole has led to drug resistance, which is the most important reason for the treatment failure[37]. If there is suspicion of metronidazole resistance, metronidazole susceptibility assay is required as it can help to determine the course of subsequent treatment[29,40]. The increase in the prevalence of metronidazole-resistant strains shows a need for development of non-nitroimidazole drugs to combat resistant trichomoniasis. Recently, some studies have been conducted to introduce new drugs against trichomoniasis. The use of herbal medicines has been considered due to fewer side effects. While the synthetic drugs are obtained from non-renewable sources as petrochemicals materials, medicinal plants, are renewable in nature[30-36,41].

#### Tinidazole

Tinidazole is antiparasitic drug from the 5-nitro-imidazole family. This drug is commonly used for treating *T. vaginalis* in America. Compared to metronidazole, tinidazole has longer half-life, higher tissue distribution, and fewer side effects. However, its mechanism of action is similar to metronidazole, and the occurrence of cross-resistance is possible. Studies have shown an incomplete cross-resistance to tinidazole in metronidazole-resistant isolates that can possibly be treated by tinidazole, but the similarity of metabolic pathways in metronidazole and tinidazole causes the rapid spread of resistance to tinidazole. Also, its cost is more than metronidazole; therefore, it cannot be used commonly[22,42].

### Literature review of herbal medicine for treatment of trichomoniasis

Active ingredients of plant extract against *T. vaginalis* are mentioned in Table 2.

**Table 2:** Active ingredients of plant extracts against *T. vaginalis* in Iran (2006-2016)

Botanical name	Active ingredients	Part used
*Artemisia aucheri Boiss.*	Artemisinin[43,51]	Leaves
*Zataria multifora Boiss.*	Thymol[44-47,51]	Leaves
*Myrtus communis*	Cineol, tannins, and flavonoids[48-51]	Leaves
*Allium sativum* (garlic)	Organosulfur compound, Allicin, Diallyl disulphide, S-Allyl cysteine, and Diallyl trisulfide[52,53,56]	Whole plant
*Ferula assa-foetida*	Monoterpenoids[54-56]	Whole plant
*Lavandula angyustifolia*	Linalool alcohol, Cineol, Camohor, Sterzoaldehyde ketone, and lavandulol[57-59]	Leaves and flowers
*Eucalyptus camaldulensis*	Terpenoid and phenolic compound[60,61]	Leaves
*Stachys sylvatica*	α-Pinene, β-Pinene, germacrene-D, and flavonoids[62,63]	Aerial parts
*Achillea millefolium*	Flavonoids[64,67]	whole plant and leaves
*Artemisia absinthium*	Monoterpene (thujone), absinthen, azulenes, phenolic compounds, Flavonoids[66,67]	Leaves
*Juglans regia*	Tannin and naphthoquinones[65,67]	Leaves and twigs
*Tanacetum Parthenium*	Tancin, lactones, and terpenes[68,69]	Leaves
*Taxus baccata*	Alkaloids (taxines A and B)[70,71]	Leaves
*Viola odorata*	Phenolic, terpenoid, and alkaloid compounds[72,73]	Leaves, flowers, and stems
*Pelarqonium roseum*	Geranium, citronellol, mannitol, and ethyl alcohol[74,75]	Leaves, flowers, and roots
*Verbascum thapsus*	Saponins, glycosides, glycosoaminoglycans, phenyl ethanol, and verbascose[76,77]	Leaves, flowers, and stems
*Allium Cepa*	Organosulfur compounds, Allyl sulfides, flavonoids, and flavenols[78,81]	Whole plant
*Oliveria Decumbens Vent*	Tymol, threpenin, and carvacrol[79]	Aerial parts
*Muscari neglectum*	Flavonoid, alkaloid, terpenoid, and steroid[80,81]	Flowers

#### Artemisia aucheri Boiss, Zataria multifora Boiss, and Myrtus communis

These plants are native in Iran and are used in traditional medicine as antiparasitic drugs. *Artemisia aucheri Boiss* contains artemisinin, which has antiparasitic properties[43], and *Zataria multifora Boiss* contains the high amount of thymol, which has anti-inflammatory, antiseptic and antibacterial effects[44-47]. *Myrtus communis* is composed of cineol, tannins and flavonoids, which have antiseptic, antioxidant, antibacterial, antiviral and antifungal activities[48-50]. Ziaiye *et al*.[51] surveyed the effect of alcoholic extract of these three plants on *T. vaginalis* *in vitro*. Their results showed that these extracts have different chemical compositions and special effects that inhibit the growth of *T. vaginalis*. Also, all of them had maximum effect in 0.1% w/v and 4 hours after culture so that after this time no parasites were found alive.

#### Garlic and Ferula assa-foetida

*Allium sativum*, is the scientific name of garlic, belongs to onion genus. Garlic is native to Central Asia and has been used over than 7000 years. Garlic is used as anticoagulant, anticommon cold, anti cancer and antiparasite[52,53]. *Ferula assa-foetida* is native to the deserts of Iran and mountains of Afghanistan and is commonly cultivated in nearby India. This plant grows in different provinces of Iran such as Khorasan, Sistan and Baluchestan, Kerman, and Fars. *Ferula assa-foetida* is used as a digestion aid, antimicrobial, abortifacient, antidote for opium, remedy for asthma and bronchitis, and fighting influenza[54,55]. Sarkari *et al*.[56] studied the effect of garlic and *Ferula assa-foetida* extract on the growth and proliferation of *T. vaginalis*. Based on the results of this study, 90% of the parasites were killed one hour after adding *Ferula assa-foetida* extract at the concentration of 2 mg/ml, but only 5% of the parasites survived after 2 hours by adding garlic extract at concentration of 0.1 mg/ml.

#### Lavandula angyustifolia

Lavandula belongs to family Lamiceae and genus Lavanduleae. This plant has traditional application as a sedative as well as an antispasmodic, antiparasitic, antibacterial, antioxidant, anticancer, antivirus, antidepressants and anti-inflammatory remedy[57,58]. Investigation of antitrichomonal activity of *Lavandula angyustifolia* essential oil *in vitro* was conducted by Ezatpur *et al*.[59]. They showed that all concentrations of lavender essential oil have good antitrichomonal effect and reduce the number of parasites; all the parasites were killed 90 minutes after exposure to 0.1% *Lavandula angyustifolia* essential oil.

#### Eucalyptus camaldulensis

Various biological effects of eucalyptus, including antibacterial, antihyperglycemic, and antioxidant activities have been reported, and it seems that the essential oils of Eucalyptus, containing terpenoid and phenolic compounds, play a major role in its biological activities[60]. Effects of various concentration of *Eucalyptus camaldulensis* extracts on *T. vaginalis* parasite in culture medium have been surveyed by Hassani *et al*.[61]. The obtained results indicated that *E. camaldulensis* extract had 80% growth inhibition in a concentration of 12.5 mg/ml after 24 h. Alcoholic extract in a concentration of 25 mg/ml showed 100% growth inhibition after 24 h, and ethyl acetate extract had 100% growth inhibition with the minimum concentration of 12.5 mg/ml 24 h after culture. Eventually, water extract in the concentration of 50 mg/ml indicated 80% and 100% growth inhibition after 48 and 72 h, respectively[61].

#### Stachys sylvatica

Stachys is one of the largest genera in the Lamiaceae plant family. It is a genus of about 300 species and a subfamily of Lamioideae. The distribution of the genus covers Europe, Asia, Africa, Australasia, and North America. This plant grows in different regions of Iran’s provinces such as Isfahan, Chaharmahal and Bakhtiari, and Lorestan. In traditional medicine, this herb is considered as anti-pain, anti-neuralgia, antioxidant, antipyretic, and appetizer[62]. Sereshti *et al*.[63] investigated the effect of aqueous and alcoholic extracts of the plant *Stachys sylvatica* on *T. vaginalis* *in vitro*. Their results indicated that aqueous and alcoholic extracts of the plant after 72 hours in medium culture TYI-S-33 had no effect on *T. vaginalis*, and metronidazole was more effective than this extract[63].

#### Achillea millefolium, Juglans regia, and Artemisia absinthium

*Achillea millefolium* is a flowering plant in the family Asteraceae. It is native to the temperate regions of the Northern Hemisphere in Asia, Europe, and North America. This plant has anti-inflammatory, antispasmodic and antioxidant activities[64]. *Juglans regia* belongs to the Juglandaceae family and Juglans genus and has tannin and naphthoquinones with potent bactericidal effects. The extract of this tree is used to treat headaches, colds, skin diseases, as well as fungal, bacterial and viral infections[65]. *Artemisia absinthium* belongs to the Asteraceae family and genus of *Artemisia*. This plant has medicinal properties, including insecticide and appetizer, as well as bactericidal, anti-inflammatory and antiparasitic effects[66]. The leaves extracts of *Achillea millefolium*, *Artemisia absinthium*, and *Juglans regia* on *T. vaginalis* was studied by Khalili *et al*.[67] *in vitro*. All three plant extracts represented anti-trichomoniasis activity after 24 hour and parasites immobility increased. However, six hours after cultivation with *Achillea millefolium*, *Artemisia absinthium*, and *Juglans regia* extracts at the concentrations of 800, 400, and 800 mg/ml, respectively, all parasites were died[67].

#### Tanacetum parthenium

*Tanacetum parthenium* (local name: fever few) belongs to *Asteraceae* family and is a native plant in Iran[68,69]. *Tanacetum parthenium*, as a traditional medicinal herb, is commonly used to prevent migraine headaches and has been used as a herbal treatment to reduce fever, arthritis, and digestive problems[68,69]. Its main compound is tannin, and others compounds include lactones (parthenolide and canine) and terpenes[68,69]. Study of the effect of hydro-alcoholic extract of *Tanacetum Parthenium* on *T. vaginalis* was conducted by Arefkhah *et al*.[69]. Alcoholic extract of this plant with concentrations of 4, 5, 8, and 10 mg/ml showed the same lethal effect on the parasite as compared to metronidazole, but concentrations of 1 and 1.25 indicated no effect[69].

#### Taxus baccata

*Taxus baccata* is a conifer native to Western, Central and Southern Europe, as well as Northwestern Africa, Northern Iran, and Southwestern Asia[70,71]. This traditional medicine plant has antibacterial, antifungal, anti-inflammatory and antitumoral activities and is used for treating malaria, rheumatism, bronchitis, and asthma[70,71]. In 2013, Zarea *et al*.[71] investigated the effects of *Taxus baccata* leaves fractions on *T. vaginalis* growth in culture medium. The component of 60% of crude extract at concentrations of 200, 300, 400, and 500 μg/ml indicated higher anti-*T. vaginalis* effects than 90% (*P*<0.05). Concentration of 200 μg/ml of the extract of 60% caused 100% inhibition, but the component of 90% indicated 60% growth inhibition[71].

#### Viola odorata

*Viola odorata* is a species of the genus Viola that is native to Europe and Asia and has been introduced to North America and Australia. In herbal medicine, *V. odorata* has been used for treating a variety of respiratory ailments, insomnia, and skin disorders[72,73]. Its compounds include phenolics, violin, and glycosides. In the leaves of this plant, analgesic components are salicylic acid glycosides[72,73]. The effects of different extracts of *Viola odorata* on *T. vaginalis* in culture medium has investigated by Salehi *et al*.[73]. The crude extract of leave (4 mg/ml), flower (4 mg/ml), and root (2 mg/ml) of *Viola odorata* showed 100% growth inhibition after 24 hours[73].

#### Pelarqonium roseum

*Pelarqonium roseum* belongs to the Geraniaceae family and *Pelargonium* genus. The major compounds of this plant are geranium, citronellol, mannitol, and ethyl alcohol[74,75]. It is used in traditional medicine as analgesic, anti-dysentery, and disinfectants as well as an anti-inflammatory, a hemostatic, and an antiseptic drug[74,75]. Fakhrie-Kashan *et al*.[75] studied the effects of aqueous and alcoholic extracts of *Pelarqonium roseum* on the growth of *T. vaginalis* *in vitro*. IC50s (inhibitory concentration, 50%) of aqueous and alcoholic extracts of *Pelarqonium roseum* on the growth of *T. vaginalis* after 24 h were 54.67 and 27.63 μg/ml, respectively[75].

#### Verbascum thapsus

*Verbascum thapsus* is a member of scorphulariaceae family. This biennial plant grows in a wide range of areas and can be two meters or more in height[76,77]. *Verbascum thapsus* contains bioactive substances such as saponins, glycosides, glycosoaminoglycans, phenyl ethanol, and verbascose[76,77] and also chemical components that reduce cyclooxygenase activity. These components have anti-inflammatory activity and germicide and skin restore effects[76,77]. *Verbascum thapsus* has also been used to treat genital and urinary tract infections and diarrhea[76,77]. Induction of apoptosis in *T. vaginalis* due to the extract of this plant have been reported by Kashan *et al*.[77]. IC50s (inhibitory concentration, 50%) of ethanolic extract of *Verbascum thapsus* and metronidazole after 24 h were 39.17 and 0.0326 μg/ml, respectively. Results of this study indicated that the percentage of apoptosis after treatment of parasites with various concentrations of *Verbascum thapsus* extract (25, 50,100, 200, and 400 μg/ml) were 20.7, 37.04, 47.5, 62.72, and 86.35 respectively[77].

#### Allium cepa, Oliveria decumbens Vent, and Muscari neglectum

The onion is a vegetable and the most widely cultivated species of the genus *Allium*. This plant grows in most parts of the world and Iran flora. It is used in traditional medicine for treatment of diseases of kidney, bladder, and prostate[78]. *Oliveria decumbens Vent* belongs to Umbeliferae family and grows in Southern Iran. It is bactericide and contains tymol, threpenin, and carvacrol[79]. *Museari negleetum* is native to the Mediterranean region[80,81]. In 2015, Fakhrieh-Kashan *et al*.[81] investigated the *in vitro* therapeutic effect of alcoholic extracts *Allium cepa*, *Oliveria decumbens Vent*, and *Muscari neglectum* against *T. vaginalis*. In this study, IC50s of *Allium cepa*, *Oliveria decumbens Vent*, and *Muscari neglectum* ethanol extracts after 24 h were 572.3, 101.8, and 329.4 μg/ml, respectively[81].

In conclusion, trichomoniasis is considered as one of the most common sexually transmitted diseases in humans[1,2]. Increasing resistance to drugs such as metronidazole leads to a serious problem; thus, new effective tactics are needed to treat this infection. The current medications and the first-line drugs for treatment of trichomoniasis are metronidazole and tinidazole; both are 5-nitroimidazole drugs approved by the US FDA. Besides, using metronidazole is limited because of its high toxicity, high doses, and development of drug resistance[30,37-39]. In order to improve the treatment of *T. vaginalis* infection, natural products could be a source of new drugs with low toxicity and high activity. Recently, many efforts have been made for the evaluation of the effects of plants on the pathogenic microorganisms that can lead to vaginal infection. Due to the diversity of Iranian medicinal plants, several studies have carried out on the *T. vaginalis* using these herbs. Unfortunately, previous studies have not used an identical procedure; therefore, we cannot exactly explain which plant extracts are more effective on the parasite. However, we can draw the conclusion that all plants with the largest effect on the parasites contain the following active compounds: cineol, tannins, flavonoid, terpenoid, phenolic ones, Artemisia, lactones, glycosides, geranium, citronium, mannitol, saponins, glycosoaminoglycans, verbascose, phenyl ethanol, tymol, threpenin, carvacrol, zingerone, paradol, gingerols, shogaols, inulin, polyacetylene, arctic aside. Until now, no herbal extract has been introduced as an approved drug for the treatment of trichomoniasis. More accurate and comprehensive studies are needed to achieve the desired results in the use of plants in trichomoniasis treatment.
